# Mental Dyspepsia

**Published:** 1893

**Authors:** J. H. Kellogg


### MENTAL DYSPEPSIA.

It is doubtless true that very many people read too much. Not that they devote too many hours to the perusal of books and newspapers, but that they endeavor to cover too much space in a given time. While they read they do not "mark, learn and inwardly digest," but bolt their mental food much as the glutton devours the material provision set before him. They rapidly skim over the matter in hand, gathering, as they think, the salient points thereof, though, in reality, they miss and reject many sweet morsels that lie between the more sensational portions. They are great—indeed, omniverous—readers, and are always ready to resort to a book or newspaper for recreation, or to "pass away the time," but rarely, or never, for securing information, except on the current events of the day.

They have "heard of" almost everything; but ask them to impart some definite knowledge concerning some subject, and it will be found that they have only heard of it.

Of real solid ideas they have but few, of glimmerings of ideas many. They hold their minds a brief and unsatisfactory epitome of the most important events in the world's history, and some of the arts and sciences; but they would be utterly unfit to teach even a child the story of our own national struggles for freedom and existence, or to tell why the days are long in summer or short in winter. The natural consequence of this unnatural mode of reading—or rather, cramming—is mental dyspepsia. The facts and arguments and illustrations that should be stored and retained in the memory, to furnish mental brawn, muscle and blood, are forced through the brain, and leave but little trace behind, save remembrance of an interminable string of words, the power and meaning of which have been lost.

This continued, the mind becomes more and more diseased, and the result is mental marasmus, which, in extreme cases, may end in extinction of the reason.

As dyspepsia is one of the most prevalent of the disorders to which the human frame is subject, so is the mental disorder the most frequent that attacks the human brain. It prevails in all classes of the reading community, though most frequently found among that semi-literary class whose education raises them a little above the ordinary level, while it does not fit them to be leaders among the world's workers and thinkers. They have learned enough to give them a great craving for more, and fancy they are adding to their stock of knowledge by taking into their brain, through the eye, the mere forms of words on the printed page.

As in the physical ailments, so in the mental, the best remedy is temperance, or abstinence, and robust exercise. If one finds himself afflicted with the complaint, he should at once begin a course of severe discipline. Let him eschew those things that have been most tempting—read the daily newspapers in moderation only, and begin to *study* whatever he reads. With his eye upon the printed page, let him master every word he sees there, and not trust to the context "to give the sense of it." Aside from that which may be his special object of study, let him choose the best authors, who will give him well cooked food, instead of the fantasies of disordered imaginations. A few months steady regimen of this sort will afford great relief, and go far toward effecting a permanent cure.

J. H. KELLOGG, M. D.

